# Safety and efficacy of tolcapone in Parkinson’s disease: systematic review

**DOI:** 10.1007/s00228-020-03081-x

**Published:** 2021-01-07

**Authors:** Carlo Alberto Artusi, Lidia Sarro, Gabriele Imbalzano, Margherita Fabbri, Leonardo Lopiano

**Affiliations:** 1 Department of Neuroscience “Rita Levi Montalcini”, University of Torino, Via Cherasco 15, 10126 Torino, Italy; 2 Department of Neurology, Martini Hospital, ASL Città di Torino, Torino, Italy; 3 Department of Neurosciences, Clinical Investigation Center CIC 1436, Parkinson Toulouse Expert Center, NS-Park/FCRIN Network and NeuroToul COEN Center, Toulouse University Hospital; INSERM; University of Toulouse 3, Toulouse, France

**Keywords:** Tolcapone, Catechol-O-methyltransferase, Parkinson’s disease, Safety, Efficacy, Liver

## Abstract

**Purpose:**

Tolcapone is an efficacious catechol-O-methyltransferase inhibitor for Parkinson’s disease (PD). However, safety issues hampered its use in clinical practice. We aimed to provide evidence of safety and efficacy of tolcapone by a systematic literature review to support clinicians’ choices in the use of an enlarging PD therapeutic armamentarium.

**Methods:**

We searched PubMed for studies on PD patients treated with tolcapone, documenting the following outcomes: liver enzyme, adverse events (AEs), daily Off-time, levodopa daily dose, unified Parkinson’s disease rating scale (UPDRS) part-III, quality of life (QoL), and non-motor symptoms. FAERS and EudraVigilance databases for suspected AEs were interrogated for potential additional cases of hepatotoxicity.

**Results:**

Thirty-two studies were included, for a total of 4780 patients treated with tolcapone. Pertaining safety, 0.9% of patients showed liver enzyme elevation > 2. Over 23 years, we found 7 cases of severe liver injury related to tolcapone, 3 of which were fatal. All fatal cases did not follow the guidelines for liver function monitoring. FAERS and EudraVigilance database search yielded 61 reports of suspected liver AEs possibly related to tolcapone.

Pertaining efficacy, the median reduction of hours/day spent in Off was 2.1 (range 1–3.2), of levodopa was 108.9 mg (1–251.5), of “On” UPDRS-III was 3.6 points (1.1–6.5). Most studies reported a significant improvement of QoL and non-motor symptoms.

**Conclusion:**

Literature data showed the absence of relevant safety concerns of tolcapone when strict adherence to hepatic function monitoring is respected. Given its high efficacy on motor fluctuations, tolcapone is probably an underutilized tool in the therapeutic PD armamentarium.

**Supplementary Information:**

The online version contains supplementary material available at 10.1007/s00228-020-03081-x.

## Introduction

Tolcapone (3,4-dihydroxy-4′-methyl-5-nitrobenzophenone) was approved in 1997 by the European Medicines Agency and in 1998 by the Food and Drug Administration as the first levodopa add-on catechol-O-methyltransferase (COMT) inhibitor for the treatment of Parkinson’s disease (PD) patients with motor fluctuations [1]. The selective and reversible inhibition of COMT exploited by tolcapone leads to a reduction of the levodopa catabolism to 3-O-methyldopa, resulting in higher availability of dopamine into the brain [2]. As a consequence, the administration of tolcapone as an adjunct to levodopa improves motor fluctuations in PD patients, allowing a significant reduction of daily time spent in Off and total daily dose of levodopa [3].

**Fig. 1:**
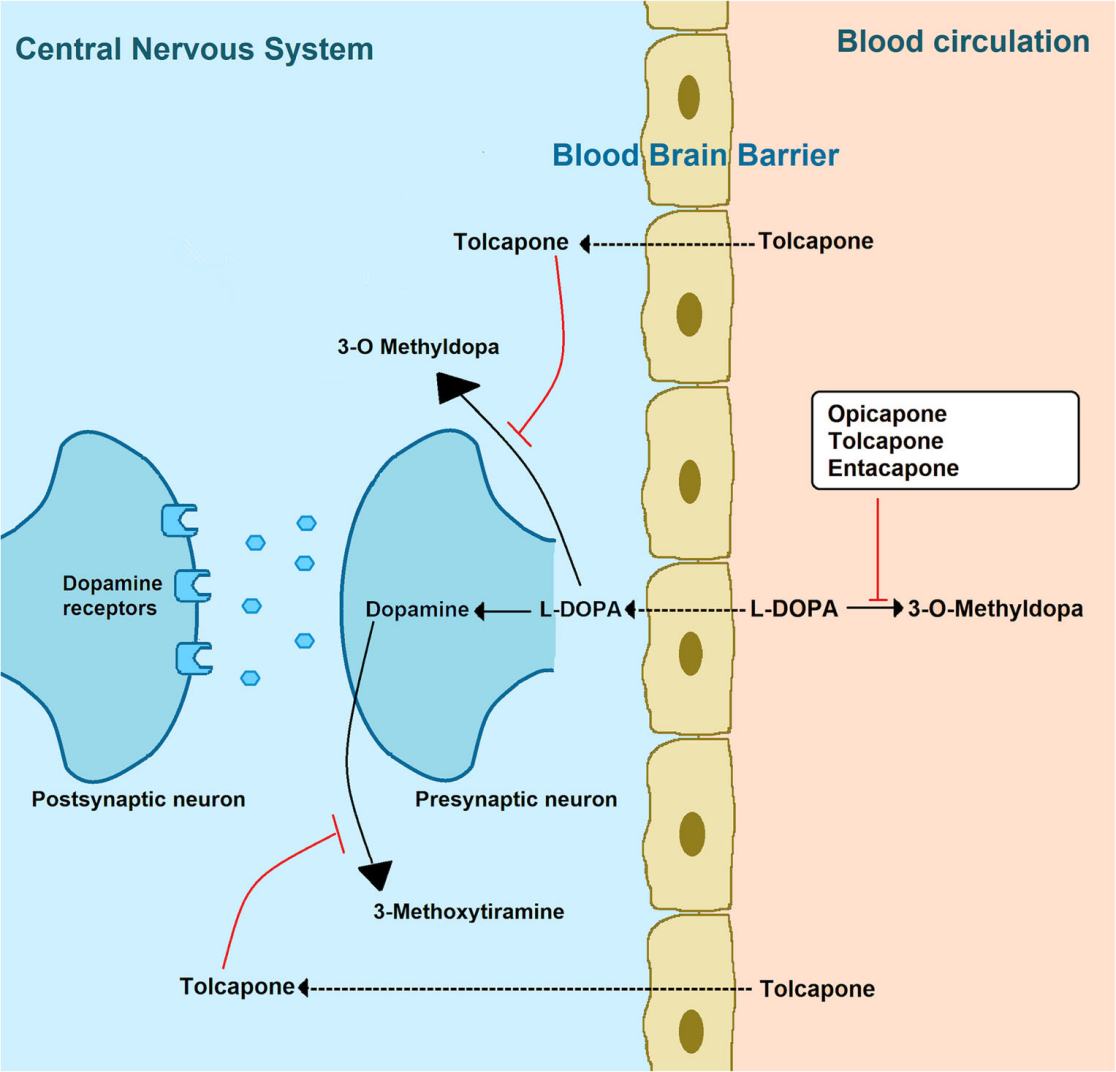
Mechanism of action of tolcapone

**Fig. 2:**
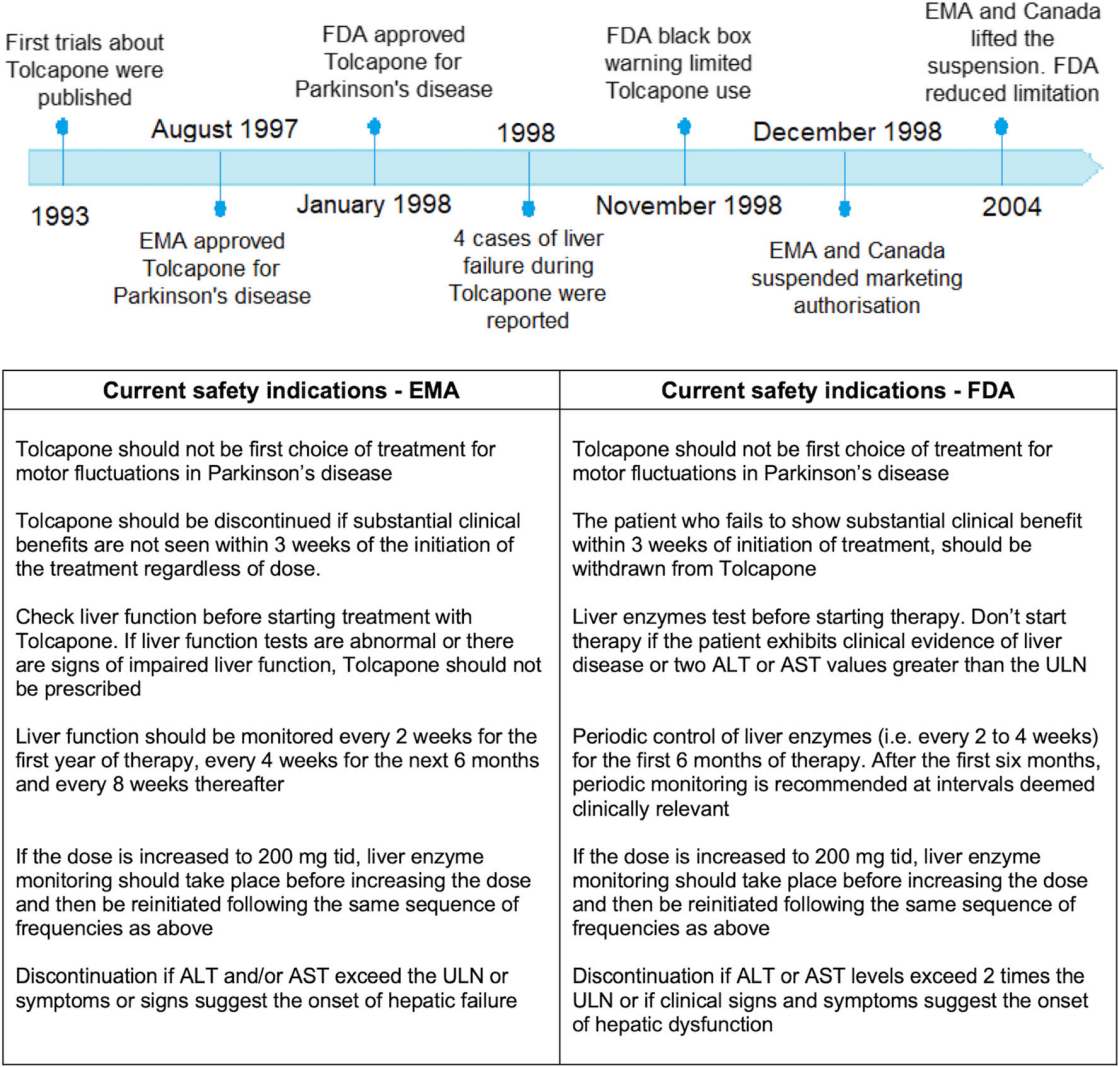
Tolcapone: history and current indications

Tolcapone differentiates from the two other COMT inhibitors available (entacapone and opicapone) for its lipophilic structure, which allows it to cross the blood–brain barrier and act into the central nervous system, exploiting its function both in the periphery and in the brain (Fig. 1) [4, 5]. Clinically, comparative data between entacapone and tolcapone showed higher efficacy of the latter [3]. Conversely, there are no studies comparing the efficacy of tolcapone and the recently marketed opicapone, although indirect data seem to indicate at least a non-inferiority of tolcapone. However, a few albeit relevant safety concerns related to the tolcapone potential hepatotoxicity have restricted its use in the clinical practice, with its prescription that should be limited to levodopa-responsive idiopathic PD patients with motor fluctuations, who failed to respond to or are intolerant of other COMT inhibitors (Fig. 2) [6] (https://www.ema.europa.eu/en/documents/product-information/tasmar-epar-product-information_en.pdf).

In this systematic review, we aimed to provide a comprehensive and updated overview of risks and potentiality linked to the use of tolcapone, beyond 20 years from its breakthrough in the PD drug armamentarium.

## Methods

### Search method

We conducted a systematic review following the Preferred Reporting Items for Systematic Reviews and Meta-analyses (PRISMA) (Supplementary Table 1) [7]. We searched PubMed for interventional and noninterventional (i.e., observational) studies prior to February 1, 2020 reporting data on patients with a diagnosis of idiopathic PD treated with tolcapone using the following searching string: “tolcapone AND Parkinson’s disease.”

Abstracts and full-text articles were independently reviewed for eligibility criteria by two authors (C.A.A., L.S.). Duplicated studies were identified and excluded. Only studies referring to human subjects and published in English were considered. No restrictions were applied to sex, age, disease duration, disease severity, or follow-up. The reference list of each article was further screened for additional pertinent studies not captured by the original search strategy.

Moreover, the “FDA Adverse Event Reporting System (FAERS)” (https://www.fda.gov/drugs/questions-and-answers-fdas-adverse-event-reporting-system-faers/fda-adverse-event-reporting-system-faers-public-dashboard) and the “EudraVigilance – European database of suspected side-effect reports” (http://www.adrreports.eu/en/index.html) databases were interrogated at May, 15th for serious adverse events (AEs) potentially related to tolcapone to search for potential additional cases of hepatotoxicity not published or not obtained by the PubMed database search.

### Study selection

We included studies assessing the effect of tolcapone as an add-on therapy to levodopa in patients with a diagnosis of idiopathic PD, and reporting data on efficacy or safety. Specifically, we included all studies reporting at least one of the following outcomes: liver enzyme, tolcapone-related AEs, daily Off-time, levodopa daily dose (mg), UPDRS part-III, non-motor PD symptoms as per validated scales, quality of life (QoL) as per validated scales. Previously published literature reviews were excluded, as well as book chapters, letters to the editor, and editorials not providing original data.

### Data extraction

Included studies were divided per study design, and for the use of a placebo or active control group when appropriate. We used a standardized data collection form to extract relevant data on safety and efficacy. The following information was extracted from each study where available: liver enzyme elevation (evaluated as the number of patients reporting an elevation of ALT or AST plasma levels), documented cases of liver failure, causes for tolcapone discontinuation, other AEs, changes of the daily Off-time, changes of the levodopa daily dose, changes of UPDRS part-III scores, score changes of validated scales for evaluating non-motor PD symptoms, score changes of validated scales for evaluating QoL in PD.

If two or more studies reported data from the same population, we included the most recent publication with the longest follow-up. Data were summarized using median and range, or percentage as appropriate. Two investigators (C.A.A., L.S.) independently performed the quality appraisal of qualifying studies. Given the heterogeneity of study designs, the risk of bias of each study was evaluated using the National Heart, Lung, and Blood Institute Quality Appraisal Tools as per the Cochrane handbook recommendations [8]. Only data from patients receiving 100 or 200 mg t.i.d. of tolcapone, the two approved dosages, were extracted and presented. When studies reported efficacy data for both dosages, only results from the most effective dosage were presented. Regarding safety data, we reported all relevant information independently from the considered dosage (100 or 200 mg t.i.d.).

### Data analysis

Results were summarized as follows: prevalence of liver enzyme elevation, number of documented cases of liver failure, prevalence of other AEs, and causes of therapy discontinuation for the safety evaluation; changes of the daily number of hours spent in Off, levodopa daily dose, UPDRS-III, QoL, and non-motor symptoms for the clinical efficacy evaluation. In studies where we found only information on the percentage reduction of hours spent in Off, we estimated the change in hours presuming 14 h of waking day.

## Results

**Fig. 3:**
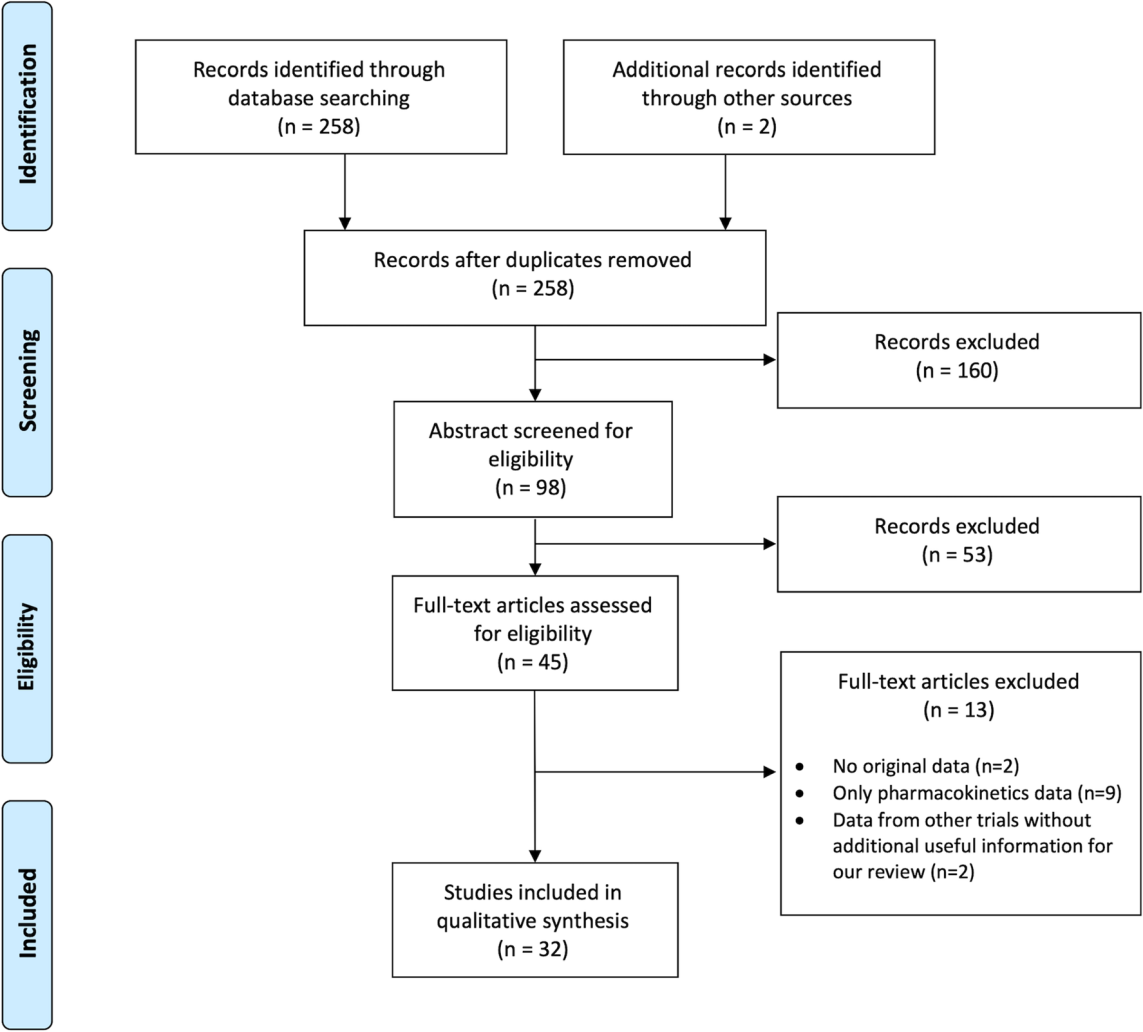
Flowchart of the systematic review

Of 258 eligible studies, 32 met full criteria (15 RCTs, 3 cross-over trials, 1 non-randomized control trial, 1 RCT post hoc analysis, 1 open-label study on a group of patients enrolled from RCT, 1 controlled before and after study, 4 before and after studies, 3 prospective cohort studies, 1 retrospective cohort study, 1 case-control study, and 1 case report) [9–40] and underwent data extraction and quality assessment (Fig. 3).

Included studies provided data from a total of 4780 patients treated with tolcapone, 4575 when considering only the dose of 100 or 200 mg t.i.d. Thirteen studies (40%) reported data on control groups treated with placebo, and six studies (18%) reported data on active control groups, treated with entacapone (*n* = 4 studies), pergolide (*n* = 1), or bromocriptine (*n* = 1).

### Safety

#### Liver enzyme elevation

A systematic evaluation of liver enzymes in patients exposed to tolcapone was reported in 21 studies (10 RCTs, 1 cross-over trial, 1 controlled before and after study, 5 before and after studies, 2 prospective cohort studies, 1 retrospective cohort study, 1 case-control study), including a total of 4181 patients treated with tolcapone, evaluated for a median follow-up of 2.3 months (range 0.5–24) (Table 1).

**Table 1:** Reviewed articles

Study ID	Study design	Patients treated with tolcapone (*n*)—dosage	Mean follow-up (weeks)	Control patients—other tolcapone dosages	Age—PD duration (year)	Off-time reduction (hours)	Total Levodopa daily dose change (mg)	UPDRS-III improvement	Liver-related adverse effects in tolcapone group	Quality assessment
Zhang 2018 [9]	RCT	41–100 mg TID	26	29 Placebo	63.5 ± 9.6–NA	NA	NA	Tremor: − 1.73 ± 3.03, muscle stiffness: − 3.71 ± 5.34, voluntary movement: − 0.41 ± 1.66	No	Poor
Ries 2010 [10]	RCT (study for evaluation of benefit in switching from DA to tolcapone)	72–100 mg TID (DA removed by day 6)	10	-	63.5 ± 9.3–11 ± 4	− 2.03	− 67	− 6.1 (OFF)	No	Fair
		78–100 mg TID (DA removed by day 23)			63.1 ± 8.3–10 ± 5	− 2.4	− 60	− 6.5 (OFF)	No	
Lees 2007 [11]	RCT	335–100 mg TID	65	342 Placebo	63 (36–78) –2 (0–8)	NA	NA	NA	92 ALT or AST > ULN, of which 6 > 3 times ULN	Fair
Entacapone to Tolcapone Switch Study Investigators 2007 [12]	RCT	75–100 mg TID	3	75 Entacapone 1200 mg/day (mean)	65.1 ± 8.9–12.3 ± 4.8	− 1.34	NA	− 3	7 Liver enzymes > ULN	Good
Koller 2001 [13]	RCT	101–100 mg TID	12	102 Pergolide mean dose 2.2 mg/day	65.0 ± 9.2–7 ± 5	− 2 to − 3	− 108.1	− 3.3	1 with ALT 28 times the ULN and AST 14 times the ULN at week 12: recovery after withdrawal	Good
Shan 2001 [14]	RCT	20–100 mg TID	6	20 Placebo	67 ± 4–10.7 ± 3.0	− 2.21	− 55 (35.1)	− 4.25	1 aspecific AST-ALT raise in an HBV carrier	Fair
Myllyla 1997 [15]	RCT	38–200 mg TID	6	34 Placebo—37 T 50 mg - 37 T 400 mg	62 ± 11–11.0 ± 4.8	− 1.54	− 79.1 (19.9)	− 5.8 (1.9)	No	Good
Tolcapone study group 1999 [16]	RCT	72–200 mg TID	8	74 Placebo	61 ± 11–9.2 ± 5	− 3.0	− 124	− 3.1 (1)	No	Fair
Adler 1998 [17]	RCT	69–100 mg TID	6	72	62 ± 12–10.5 ± 4.8	− 2 (0.3)	− 185.5 (20.6)	− 2.3 (0.7)	No	Good
		74–200 mg TID			61 ± 10–10.5 ± 4.6	− 2.5 (0.3)	− 251.5 (19.9)	− 2.4 (0.7)	No	
Hauser 1998 [18]	RCT (8 weeks of tolcapone, with 4 weeks of Selegiline associated)	42–200 mg TID	First 4 (only tolcapone)	41 Placebo	66 ± 8–0.9 ± 1	NA	NA	− 0.6 (0.5)	1 AST and 1 ALT raises (values NA)	Good
			Second 4 (tolcapone + selegiline)			NA	NA	− 0.9 (0.6)	No	
Rajput 1998 [19]	RCT	69–100 mg TID	13	66 Placebo	63 ± 9–11 ± 5.4	− 2.3	− 166.3 (22.3)	− 1.9 (0.9)	3 aspecific liver enzymes raise	Good
		67–200 mg TID			64 ± 9–11.1 ± 5.4	− 3.2	− 207.1 (22.6)	− 2.0 (0.9)	Liver enzymes raise: 1 aspecific, 1 three to five times ULN causing withdrawal, but asymptomatic	
Baas 1998 [20]	RCT	60–100 mg TID	13	58 Placebo	62 ± 10–9.0 ± 5.0	− 2.1	− 108.9 (23.4)	− 4.2 (1.0)	1 Highly abnormal ALT and high AST raises	Fair
		59–200 mg TID			63 ± 9–10.0 ± 4.8	− 1.6	− 122.2 (23.9)	− 6.5 (1.0)	2 Highly abnormal ALT and high AST raises, with 1 withdrawal	
Waters 1997 [21]	RCT	98–100 mg TID	26	102 Placebo	67 ± 9–4.2 ± 2.5	NA	− 20.8 (9.7)	− 2.0 (0.6)	3 aspecific liver enzymes raise, with 1 withdrawal	Good
		98–200 mg TID			63 ± 11–3.4 ± 2.0	NA	− 32.3 (9.6)	− 2.3 (0.6)	5 aspecific liver enzymes raise, with 3 withdrawal	
Kurth 1997 [22]	RCT	40–200 mg TID	6	42 Placebo—41 T 50 mg–38 T 400 mg	65 ± 8–8.5 ± 6.42	− 1.3	− 200 (31)	− 37 (8.9) in AUC of 30 min intervals evaluations in 10-h	No	Good
Dupont 1997 [23]	RCT	32–200 mg TID	6	33 Placebo—32 T 400 mg	66 ± 9–NA	NA	− 182.0 (23.4)	− 3.4 (1.3)	No	Good
Factor 2001 [24]	Non-randomized controlled trial	14–200 mg TID	52	9 Entacapone 800–2000 mg/day	66 - NA	− 1.0 (0.23)	− 218.2 (77.3)	− 2.6 (2.2)	No	Poor
Welsh 2000 [25]	Post-hoc analysis RCT	12–200 mg TID	6	14 Placebo—10 T 50 mg–10,400 mg	64–9	NA	NA	NA	No	Fair
Gasparini 1997 [26]	RCT subgroup analysis	8–200 mg TID	26	-	64 ± 5.6–12 ± 7.3	NA	NA	− 4.5 (OFF) − 9.37 (ON)	No	Poor
Muhlack 2014 [27]	Cross-over Trial	22–100 mg TID	450 min	-	63.96 ± 7.1–NA	NA	NA	NA	No	Fair
Onofrj 2001 [28]	Cross-over Trial	40–100 mg TID	3–7 months	-	64 ± 6–10.4	− 2.6	− 186 (22)	− 4.3 ± 2.1	2 withdrawal: one had liver enzymes 4 times ULN, one 3 times ULN	Fair
Limousin 1995 [29]	Cross-over Trial	10–200 mg TID	Hours	-	68 ± 2–13 ± 1	NA	NA	NA	No	Poor
Meco 2000 [30]	Controlled before and after study	7–NA	26	-	69.7 (58–81)–14.1 (10–25)	NA	NA	NA	No	Poor
Müller 2014 [31]	Before and after	125–100 mg TID	4	-	70 ± 7.8–9.7 ± 5.9	− 1.62	NA	NA	2 AST aspecific raise, 2 ALT aspecific raise	Fair
Sethi 2010 [32]	Before and after	192–100 mg TID	30 days	-	NA	NA	NA	NA	No	Poor
Canesi 2008 [33]	Before and after	66–100 mg TID	52	-	64.0 ± 9.7–15.9 ± 5.7	− 1.27	− 107.1	− 1.1	No	Fair
Suchowersky 2001 [34]	Before and after	116–100 mg TID	4	-	63 (1.4)–4.5	NA	− 49	− 3.6	No	Good
Eggert 2014 [35]	Prospective cohort	391–100 mg TID	52	-	67.3 ± 9.4–11.9 ± 5.8	NA	NA	NA	29 aspecific liver enzymes raise, 3 ALT raises > 2 times ULN and 2 AST raises > 8 times ULN	Fair
Ebersbach 2010 [36]	Prospective cohort	61–100 mg TID	30.8 ± 7.5 days	-	68.3 ± 7.2 (45–82) –11.4 ± 5.8 (2.7–36.3)	− 1.8	NA	NA	1 AST aspecific raise	Fair
Ebersbach 2009 [37]	Prospective cohort	237–100 mg TID	159.4 ± 97.7 days	-	69.9 ± 8.2 (47–90)–9.5 ± 5.0 (1–25)	NA	NA	NA	18.4% of patients had aspecific liver enzymes raise	Fair
Lew 2007 [38]	Retrospective cohort	1725–100 mg TID or 200 mg TID	104	-	NA–NA	NA	NA	NA	65 aspecific liver enzymes raise	Fair
Rojo 2001 [39]	Case-control	8–100 mg TID	2	15	60.4 ± 4.6–NA	NA	− 0.8	NA	1 liver enzymes raise (values NA)	Poor
Assal 1998 [40]	Case report	1–100 mg BID	9	-	74–20	NA	NA	NA	Liver failure at 9 weeks of treatment, death after 14 days	-

Where applicable, data are means ± SD or (SEM), or (range)

Where applicable, mean follow-up is reported in weeks

UPDRS-III, were not indicated, was calculated during the ON-time

Off-time reduction, were not specifically reported in hours, is calculated as estimated ((% of waking day × 14 h)/100)

*PD*, Parkinson’s disease; *RCT*, randomized clinical trial; *BID*, twice a day; *TID*, three times a day; *DA*, dopamine agonist; *AST*, aspartate aminotransferase; *ALT*, alanine aminotransferase; *ULN*, upper limit of normal; *T*, tolcapone; *HBV*, hepatitis B virus; *NA*, not available; *AUC*, area under the curve

Eighty-one percent of studies (*n* = 17/21) searched for liver enzyme elevation during treatment with tolcapone, with percentage of patients presenting liver enzyme elevation ranging from 0 to 27.5%. In the vast majority of cases, the enzyme elevation was reported as mild and “aspecific,” while 0.9% of patients (*n* = 36) showed an elevation of liver enzymes > 2 upper limit normal. A total of 0.6% of patients (*n* = 23) receiving tolcapone withdrew from studies because of liver enzyme elevation.

Thirteen studies reported data on liver enzyme also in control groups of PD patients not treated with tolcapone. Twenty-three percent of these studies (*n* = 3/13) reported cases of liver enzyme elevation. All three studies had an active control group treated with entacapone (*n* = 2) or pergolide (*n* = 1). The percentage of controls reporting a liver enzyme elevation ranged from 0 to 20.2%.

#### Liver failure

No cases of liver failure related to tolcapone administration were reported in RCTs, nor in other observational studies on tolcapone yielded by our systematic review. We found four cases reported in 1998 on liver failure in PD patients treated with tolcapone, with no antecedents of liver dysfunction, which led to the marketing suspension of tolcapone [41]. Only one of these cases was retrieved by our PubMed research as a case report [40], while the others were indirectly reported in a manuscript published by the Tasmar Advisory Panel [41]. A case of fulminant hepatitis has been described in a 74-year-old woman and a disease duration of 20 years, with tolcapone 100 mg/day bid. Concomitant antiparkinsonian treatment was levodopa/benserazide 100 mg/25 mg t.i.d. She developed a severe liver failure in 9 weeks. Despite discontinuation of tolcapone, she rapidly deteriorated and she died in hepatic coma 14 days after admission, about 1 month after symptom onset. The second case of liver failure occurred in a 73-year-old woman, taking tolcapone 200 mg t.i.d. and levodopa/carbidopa (dose not available). She had several comorbidities including aortic regurgitation, septicaemia due to a staphylococcus infection of the back, weight loss, and depression. She developed liver failure 12 weeks after starting tolcapone, but tolcapone was not discontinued and she died 2 weeks later. Similarly, another 74-year-old woman, taking levodopa/carbidopa 100/25 mg, 8 times/day, developed a liver failure 11 weeks after starting tolcapone 100 mg t.i.d. Tolcapone was halted after 1 week since the onset of symptoms, but few days later she had an episode of coffee-ground emesis in the context of a cirrhosis. She developed an acute distress respiratory syndrome treated with fresh frozen plasma, but she deceased in few days. Finally, a fourth case of liver dysfunction has been reported in a 66-year-old woman, under tolcapone 200 mg t.i.d. for about 3 months. She was hospitalized 2 weeks after symptom onset, tolcapone was halted and liver enzymes significantly decreased in 3 days. The liver biopsy did not find evidence of cirrhosis. She was treated with vitamin K, fresh frozen plasma, and prednisone, recovering in a week.

Beyond the abovementioned four clinical cases described in 1998, our systematic review could not find other cases of hepatic fatality. However, other three cases of severe liver injury possibly related to tolcapone were summarized in a safety review of tolcapone in 2007 [42].

The interrogation of the FAERS and EudraVigilance databases for suspected serious AEs during the treatment with tolcapone yielded 61 reports (14 from EudraVigilance and 47 from FAERS) of hepatitis, liver failure, liver toxicity, or jaundice from 1997 to May 15, 2020. Among these, 19 reports described the co-occurrence of major disease or syndromes, such as neuroleptic malignant syndrome, multiple organ failure, neoplasm, shock, sepsis, complicated mononucleosis, and in one case congenital absence of bile ducts. Seven of these reports resulted in death, 10 recovered or had minor sequelae, and 2 cases had unknown outcome. The remaining 42 suspected AEs reports (8 from EudraVigilance and 34 from FAERS) showed fatality in 9 cases, unknown outcome in 3 cases, while 30 cases resolved or remained stable; no further detail about sequalae was available. Half of reported cases occurred early in 1998 (Table 2).

**Table 2:** Adverse events during tolcapone available on FDA and EUDRA reporting system

*N* of cases	61
Cases from FDA database	14
Cases from EUDRA database	47
Year of event	
1998	31
1999	7
2003	1
2004	1
2005	1
2006	2
2007	2
2008	3
2009	2
2010	1
2011	1
2012	1
2013	5
2016	2
2017	1
Age	
18–64	18
> 65	40
Not specified	3
Sex	
Male	29
Female	32
Patients with concomitant serious disease*	20
Suspected product	
Tolcapone	39
Tolcapone + benserazide/levodopa	7
Tolcapone + ropinirole	3
Tolcapone + benserazide/levodopa + pergolide	2
Tolcapone + levodopa/carbidopa+ apomorphine + ropinirole	1
Tolcapone + other drugs	9
Reported liver damage	
Severe**	31
Non-severe***	30
Outcome at time of last reporting	
Hospitalization	18
Other outcomes	15
Death ****	
1998	10
≥ 1999	6
Recovered	6
Unknown	3
Not recovered/not resolved	2
Required Intervention	1

*Concomitant serious disease: neuroleptic malignant syndrome; congenital absence of bile ducts; intestinal infarction; rhabdomyolysis; shock; cardiac failure; cancer; cirrhosis; renal failure; mononucleosis; non-Hodgkin’s lymphoma; sepsis; infection; medication error; pancreatitis; hepatorenal failure; pleural fibrosis; stroke; atelectasia; pneumonia

**Severe liver damage reported: hepatitis fulminant; hepatitis; acute hepatic failure; hepatic failure; hepatic necrosis; hepatic encephalopathy; hepatic cirrhosis; portal hypertension; hepatorenal failure; biliary cirrhosis

***Non-severe liver damage reported: jaundice; cholestatic liver injury; liver function test abnormal; hepatotoxicity; alanine aminotransferase increased; aspartate aminotransferase increased; blood bilirubin increased; blood alkaline phosphatase increased; gamma-glutamyltransferase increased; hepatic enzyme increased; drug-induced liver injury; hepatomegaly; cholelithiasis; liver disorder; hepatic steatosis; cholecystitis; hyperammonaemia

****Of the 16 death patients, 10 had concomitant serious disease, and 1 was positive for antimitochondrial antibodies

#### Causes of tolcapone discontinuation and other adverse events

Twenty-nine studies reported data on AEs (all but 1 retrospective cohort study, 1 RCT post hoc analysis, 1 open-label study, on a group of patients enrolled from RCT), for a total of 2748 patients treated with tolcapone and evaluated for a median follow-up of 2.5 months (range 0.5–15) (Table 1). The most common AEs reported were liver enzyme elevation (ranging from 0 to 27% of patients), diarrhea (from 0 to 29%), urine discoloration (from 0 to 23%), and dopaminergic symptoms, such as dyskinesia (from 1 to 95%), hallucinations (from 0 to 24%), nausea (from 0 to 68%), and dizziness (from 0 to 16%). Analyzing AEs in studies comparing the effect of tolcapone with an active control group, there was no significant difference in occurrence and types of AEs between tolcapone and entacapone groups (*n* = 2 studies); there was a higher proportion of AEs related drops-out in the pergolide group; and there was a higher incidence of nausea, orthostatic complaints, hallucinations, and peripheral edema in the bromocriptine group vs. tolcapone, but lower incidence of muscle cramps, dystonia, and xerostomia.

Excluding case reports, 21 studies reported data on patients withdrawing tolcapone with related causes. From studies reporting data on drop-out, a total of 10.3% of patients (*n* = 270/2631) withdrew tolcapone due to AEs. The most common causes for tolcapone discontinuation were: diarrhea, liver enzyme elevation, nausea, and dopaminergic symptoms. No cases of liver failure were found, with the exception of one patient with liver metastasis from breast cancer.

Diarrhea was reported in 12 studies as an AE occurring during tolcapone treatment. From these studies, 6.8% of patients (*n* = 94/1384) withdrew tolcapone because of diarrhea. Seven out of 12 studies reported AE data from a control group, showing 0.9% (*n* = 6/666) of control drop-out due to diarrhea.

### Efficacy

#### Reduction of daily time spent in Off

Twelve studies reported data on daily hours spent in Off (9 RCTs, 1 cross-over trial, 1 before and after study, 1 prospective cohort study), for a total of 967 patients treated with tolcapone evaluated for a median follow-up of 1.7 months (range 0.7–7) (Table 1). The median reduction of hours/day spent in Off was 2.1, ranging from 1 to 3.2. Whenever applicable, 100% of studies reported a significant reduction of the Off hours after administration of tolcapone (within group difference). Twelve studies reported data on daily Off change comparable with a group of patients in levodopa plus placebo (*n* = 7) or levodopa plus bromocriptine (*n* = 1), pergolide (*n* = 1), or entacapone (*n* = 3) (between groups difference). All but one studies vs. placebo found a significant difference between patients treated with tolcapone and the control group. Two out of three studies vs. entacapone reported a significant higher reduction of Off hours in the tolcapone group, while the 2 studies comparing tolcapone with dopamine-agonists pergolide and bromocriptine found a similar extent of improvement.

#### Reduction of levodopa daily dose

Sixteen studies reported data on levodopa daily dose (11 RCTs, 1 cross-over trials, 1 non-randomized control trial, 2 before and after studies, 1 case-control study), for a total of 995 patients treated with tolcapone evaluated for a median follow-up of 2.5 months (range 0.5–12) (Table 1). The median reduction of levodopa was 108.9 mg, ranging from 1 to 251.5 mg. Six studies reported a significant reduction of levodopa dose after tolcapone starting. Twelve studies reported data on levodopa daily dose comparable with a group of patients in levodopa plus placebo (*n* = 8) or levodopa plus bromocriptine (*n* = 1), pergolide (*n* = 1), or entacapone (*n* = 2). Seventy-five percent of studies vs. placebo (*n* = 6/8) found a significant lower levodopa daily dose between patients treated with tolcapone and the control group. The two studies vs. entacapone and the study vs. bromocriptine reported a significant higher reduction of levodopa in the tolcapone group, while the study comparing tolcapone with pergolide found a similar extent of levodopa dose reduction.

#### Improvement of UPDRS motor score

Seventeen studies reported data on UPDRS-III changes over time (12 RCTs, 1 cross-over trials, 1 non-randomized control trial, 1 open-label study on a group of patients enrolled from RCT, 2 before and after studies), for a total of 1113 patients treated with tolcapone evaluated for a median follow-up of 2.2 months (range 0.7–12) (Table 1). A percentage of 64.7 of studies (*n* = 11/17) reported the UPDRS-III score in the On condition, 11.8% (*n* = 2/17) in both the Off and On conditions, and 5.9% (*n* = 1/17) in the Off condition; 17.6% of studies (*n* = 3/17) did not specify whether UPDRS-III scores were in the Off or On condition. The median reduction of UPDRS-III in On was − 3.6 points, ranging from − 1.1 to − 6.5. Five studies evaluated whether the UPDRS-III change between pre and post tolcapone administration was significant, and all but one of these studies found a significant improvement after tolcapone administration, after a median follow-up of 5 months (range 1–12). One additional study evaluating “acute” UPDRS-III score changes till 420 min after the administration of tolcapone found a significant UPDRS-III improvement [27].

Fourteen studies reported data on UPDRS-III score changes comparable with a group of patients in levodopa plus placebo (*n* = 8) or levodopa plus bromocriptine (*n* = 1), pergolide (*n* = 1), or entacapone (*n* = 4). Thirty-seven percent of studies vs. placebo (*n* = 3/8) found a significant difference between patients treated with tolcapone and the control group in favor of tolcapone patients. The studies analyzing the tolcapone group vs. an active control group did not find significant difference in the UPDRS-III score changes.

#### Improvement of QoL

Nine studies reported data on QoL (5 RCTs, 1 RCT post hoc analysis, 2 before and after studies, 1 prospective cohort study), for a total of 801 patients treated with tolcapone evaluated for a median follow-up of 2.5 months (range 1–6). There was heterogeneity on the assessment of QoL: 5 studies evaluated QoL changes by the Sickness Impact Profile (SIP), 2 studies by the EuroQol-5D, 1 study by the Parkinson’s disease questionnaire 8 (PDQ-8), and 1 study by both the SIP and the PDQ-39. Independently on the type of assessment, 80% of studies reporting the information (*n* = 4/5) found a significant improvement of QoL after administration of tolcapone. Five studies reported data on QoL changes comparable with a group of patients in levodopa plus placebo (*n* = 4) or levodopa plus pergolide (*n* = 1). Fifty percent of studies vs. placebo (*n* = 2/4) found a significant difference between patients treated with tolcapone and the control group in favor of tolcapone patients, and also the study comparing tolcapone vs. pergolide found a significantly higher improvement of QoL in patients treated with tolcapone.

#### Improvement of non-motor symptoms

Ten studies reported data on non-motor symptoms (6 RCTs, 1 cross-over trials, 1 open-label study on a group of patients enrolled from RCT, 1 before and after study, 1 prospective cohort study), for a total of 554 patients evaluated for a median follow-up of 3 months (range 1–7). There was heterogeneity on the assessment of non-motor symptoms: only 1 study employed a specific scale validated for the assessment of non-motor symptoms, the “Non-motor Symptoms Questionnaire” (NMSQ) [31]. Eight studies reported non-motor data by UPDRS-I score, 1 study employed specific scale to assess the sleep quality (Parkinson’s disease sleep scale (PDSS), and Epworth sleepiness scale (ESS)) [36], and 1 study analyzed differences in cognitive functions by means of a comprehensive battery of neuropsychological tests [26].

The study analyzing non-motor symptoms by NMSQ and UPDRS-I found a significant improvement of patients 1 month after starting tolcapone in both scales, with a mean reduction of 2.5 points for the NMSS and 1 point for the UPDRS-I. The 2 other studies, using UPDRS-I and reporting information on significant difference before and after tolcapone, did not report a significant improvement. The study assessing the sleep reported a significant improvement of both PDSS and ESS, with a mean score reduction of − 15.3 and − 1.3, respectively. The study assessing cognitive functions of 8 PD patients at baseline and after 6 months of treatment with tolcapone found improvement in attentional task, auditory verbal short-term memory, visuo-spatial recall, and constructional praxia.

Five studies reported data on non-motor symptom changes comparable with a group of patients in levodopa only and no studies compared this outcome with active control groups. Twenty percent of studies (*n* = 1/5) found a significant higher improvement of symptoms (as per UPDRS-I score) than placebo group.

## Discussion

We performed a systematic review of studies reporting clinical data on the safety and efficacy of tolcapone as an adjunct therapy to levodopa at the dosage of 100 or 200 mg t.i.d. We found that from 21 studies, of which 10 RCTs, reporting data of 4181 tolcapone treated patients, an elevation of mild liver enzymes was frequent (ranging from 0 to 27% of patients), but elevation > 2 upper limit normal was reported in < 1% of patients. Three RCTs comparing efficacy and safety of tolcapone with other add-on therapies (entacapone in 2 studies and pergolide in 1 study) reported elevation of liver enzymes also in the control group. Three cases of fatal liver failure and one case of severe reversible hepatotoxicity were reported in 1998 [40, 41]. Noteworthy, none of these patients had followed monitoring guidelines, and in one case tolcapone was not withdrawn even after the development of clinical evidence of hepatic failure [41]. After these cases, in the last 20 years we could find only three, not fatal cases of severe liver injury possibly related to tolcapone [42] and no other studies on PubMed reporting relevant safety issues with tolcapone. However, we retrieved 61 further reports of severe AEs involving the liver registered in the EMA and FDA databases for post-authorization drug surveillance. The interpretation of data extracted from these databases requests important caveats. First, the causality link between the drug and the reaction cannot be deduced from data since the report reflects only a subjective observation by the reporter. Furthermore, the submission of a report does not undergo to formal revision or medical check, the reporter could be a non-healthcare professional, data could be incomplete, and information could be duplicated if more than one reporter independently submit the same case. Nevertheless, these data disclose precious information concerning possible adverse reactions that otherwise would be lost.

Excluding the hepatic concern, the safety profile of tolcapone can be considered comparable to other COMT inhibitors, including a low drop-out rate for dyskinesia, being the most frequent cause of treatment discontinuation in patients under opicapone 50 mg/day [43].

Concerning the efficacy, the median reduction of hours spent in Off was 2.1, which is significant in the vast majority of studies also compared to placebo or entacapone. Such a data is consistent with the Cochrane meta-analysis on add-on levodopa treatment effect, which reports on an Off-time reduction of − 1.6 h/day (CI 95% − 2.0 to − 1.2) for tolcapone [3]. These values place tolcapone as the most effective COMT inhibitor in terms of motor fluctuations, if compared to entacapone and the recently marketed opicapone, providing an Off-time reduction of about − 0.61 and − 1 h/day, respectively [44]. While direct comparisons between tolcapone and entacapone showed the higher efficacy of the former, the superiority of tolcapone on opicapone should be better supported by a head-to-head trial comparison.

Data on motor fluctuations and dyskinesia provided by UPDRS-IV was fully available only in 2 out of 32 studies and thus we could not provide a reliable analysis of this outcome. However, the great efficacy of tolcapone is further supported by a median reduction of levodopa of 108.9 mg, which was higher than patients treated with entacapone and bromocriptine in three studies, and by a UPDRS-III score in On improved of a median of − 2.8 points, which can be considered a clinically meaningful change [45]. Moreover, QoL significantly improved in 50% of studies comparing tolcapone versus placebo, and in one study comparing tolcapone vs. pergolide. Finally, studies investigating non-motor symptoms found efficacy of tolcapone on total NMSQ and UPDRS-I scores, although drawing conclusions on the effect of tolcapone on single non-motor symptoms is not possible. Results from one study suggested a sleep improvement as per the significant improvement of both PDSS and ESS scale scores, and another study found a global cognitive improvement of patients treated with tolcapone [26, 36]. The improvement of these specific non-motor symptoms might be related to the concomitant reduction of motor fluctuations and the improvement of parkinsonian symptoms.

After more than 20 years from its first approval and about 14 years after its reintroduction into the market, we have a deeper knowledge of the tolcapone safety profile, which allows clinicians to safely manage and properly monitor possible liver AEs. Even if tolcapone is still considered a second-line levodopa add-on treatment [6], mainly due to past safety concerns, we have observed that liver enzyme elevation, which can be frequent, is considered mild or aspecific in about 99% of cases. Indeed, a very low number of patients developed liver injuries after tolcapone remarketing with the new guidelines for liver function monitoring (Fig. 2) (https://www.ema.europa.eu/en/documents/product-information/tasmar-epar-product-information_en.pdf). A multi-center, observational study on 391 patients treated with tolcapone under routine practice conditions, showed that tolcapone is safe in PD patients following the guidelines for monitoring liver enzymes, proving that significant liver transaminase elevations were rare and generally returned to normal without intervention in most patients [35]. Moreover, Lees and coll. [11] analyzed the safety and tolerability profile of tolcapone enrolling 667 levodopa-naive patients with early-stage PD and randomized to receive placebo or tolcapone 100 mg t.i.d, added to standard doses of levodopa; they found liver values above the upper limit normal in 20.2% of patients receiving levodopa plus placebo and 27.5% in the tolcapone group, including those with increased values at screening; increases 3 times the upper limit normal occurred in 1.8% of tolcapone treated patients and 1.2% of placebo treated patients (the difference was not statistically significant).

This systematic review has two main shortcomings that should be taken into account when interpreting the results. First, we limited our search to the PubMed database, and some pertinent studies, including gray literature, could have been missed. Second, we did not apply a meta-analytic data analysis. This aspect should be especially considered when interpreting comparisons of data between tolcapone and other COMT inhibitors.

Limitations notwithstanding, available evidence indicates that tolcapone is a highly effective add-on therapy for PD, without relevant safety concerns when adherence to the FDA and EMA prescription guidelines is respected.

## Supplementary Information

ESM 1 (DOCX 17 kb)

## Data Availability

All data used for this review are provided in the main text and tables. Sources of data are referenced in the appropriate section.
